# Joint Estimation for Time Delay and Direction of Arrival in Reconfigurable Intelligent Surface with OFDM

**DOI:** 10.3390/s22187083

**Published:** 2022-09-19

**Authors:** Jinzhi Du, Weijia Cui, Bin Ba, Chunxiao Jian, Liye Zhang

**Affiliations:** National Digital Switching System Engineering & Technological Research Center, Zhengzhou 450001, China

**Keywords:** tensor, time delay (TD), direction of arrival (DOA), reconfigurable intelligent surface (RIS), channel frequency response, joint estimation

## Abstract

Recently, the joint estimation for time delay (TD) and direction of arrival (DOA) has suffered from the high complexity of processing multi-dimensional signal models and the ineffectiveness of correlated/coherent signals. In order to improve this situation, a joint estimation method using orthogonal frequency division multiplexing (OFDM) and a uniform planar array composed of reconfigurable intelligent surface (RIS) is proposed. First, the time-domain coding function of the RIS is combined with the multi-carrier characteristic of the OFDM signal to construct the coded channel frequency response in tensor form. Then, the coded channel frequency response covariance matrix is decomposed by CANDECOMP/PARAFAC (CPD) to separate the signal subspaces of TD and DOA. Finally, we perform a one-dimensional (1D) spectral search for TD values and a two-dimensional (2D) spectral search for DOA values. Compared to previous efforts, this algorithm not only enhances the adaptability of coherent signals, but also greatly decreases the complexity. Simulation results indicate the robustness and effectiveness for the proposed algorithm in independent, coherent, and mixed multipath environments and low signal-to-noise ratio (SNR) conditions.

## 1. Introduction

Reconfigurable, intelligent surface (RIS) is a planar array consisting of reflective elements equipped with a time-domain encoding that can alter the frequency characteristics of incoming signals [1]. At present, the RIS has been widely researched in the fields of wireless communication [2], beamforming [3], and direction of arrival (DOA) estimation [4]. The RIS has the function of time-domain encoding, which can change the frequency characteristics of received signals [1]. Orthogonal frequency division multiplexing (OFDM) is a multi-carrier digital modulation technology which utilizes multiple parallel subcarriers to realize serial high-speed data communication. OFDM provides data transmission and positioning services for users and is widely used in wireless local area networks (WLAN) [5], 5G mobile communications [6], and time delay (TD) estimation [7]. A reasonable combination RIS functions and OFDM features can improve performance in a variety of wireless applications [8,9,10].

TD and DOA are important components of positioning systems, such as for indoor localization [11] and radar [12]. Like the time-domain narrowband signal model, the frequency-domain DOA estimation algorithm is studied by the multiple signal classification (MUSIC) method [13]. However, the capability of this algorithm is restricted to the size of the array aperture. An Algorithm [14] is used for TD estimation of OFDM signals, but it is not available in multipath environments. As researchers choose super-resolution methods such as the propagator method (PM) [15], MUSIC [16] and estimating signal parameters via the rotational invariance techniques (ESPRIT) [17], it is difficult to improve the accuracy of the estimations due to signal bandwidth constraints.

The joint estimation for TD and DOA by space–time parameter coupling not only improves the estimation performance, but also reduces the number of receiver nodes and improves the efficiency of the location system. Thus, the joint estimation has obvious strengths. Some researchers proposed a method [18] to solve TD under wideband signal conditions and then estimate DOA based on the trigonometric geometry TD inequality, but its performance does not improve significantly. In addition, a new method [19] was proposed to provide the frequency response of extended channels by using a Hadamard product, which achieves high accuracy joint estimation on OFDM systems. Nevertheless, the complexity of this approach is extremely high due to the demand for a full-field search.

The above algorithms are performed in an independent environment, which is necessary for the subspace approaches. For the coherent signal estimation, an approach [20] employing cyclostationary signals and a method [21] for separating coherent signal components have been proposed, both of which lead to more complex systems that are hard to implement in a joint estimation model. In addition, the method in [22] applies smoothing preprocessing to joint estimation to improve the adaptability in a coherent environment. However, smoothing preprocessing results in aperture loss and limited estimation accuracy.

The main efforts and results of this paper are summarized in the following points.

(i) We present a joint estimation algorithm for TD and DOA. The algorithmic model integrates the RIS array response and the OFDM subcarrier response to build a coded channel frequency response (Coded-Response). The algorithm achieves excellent estimation performance under low signal-to-noise ratio (SNR) conditions.
(ii) Due to the reasonable combination of the RIS time-domain coding function and the OFDM multi-subcarrier features, we construct frequency asymmetry of the space–time phase. This method reduces the singularity of the signal correlation matrix and thus effectively suppresses the coherence signal.
(iii) The covariance matrix of Coded-Response is reconstructed by using the structural advantages of tensor. Further use of CANDECOMP/PARAFAC to decompose the covariance matrix results in corresponding TD and DOA signal subspaces, avoiding multi-dimensional spectral peak search and greatly reducing complexity.
(iv) Compared with the existing RIS- and OFDM-based localization algorithms [23,24], the proposed algorithm can obtain the required parameters for localization based on a single station node in a coherent environment. Simulation results show that the proposed algorithm avoids the aperture loss of current smoothing algorithms [22,25] when processing coherent signals and thus has more advantages in terms of estimation accuracy.

The rest of the paper is summarized below. First, we describe the details of the signal model in Section 2. In Section 3, we describe the joint estimation algorithm under tensor structure and list the steps of the proposed algorithm. We perform computational complexity analysis in Section 4. For Section 5, we analyze the simulation performance. Finally, we summarize our efforts in Section 6. The notations used in this paper are described in Table 1.

## 2. Signal Model

### 2.1. Time-Domain Model

**Figure 1:**
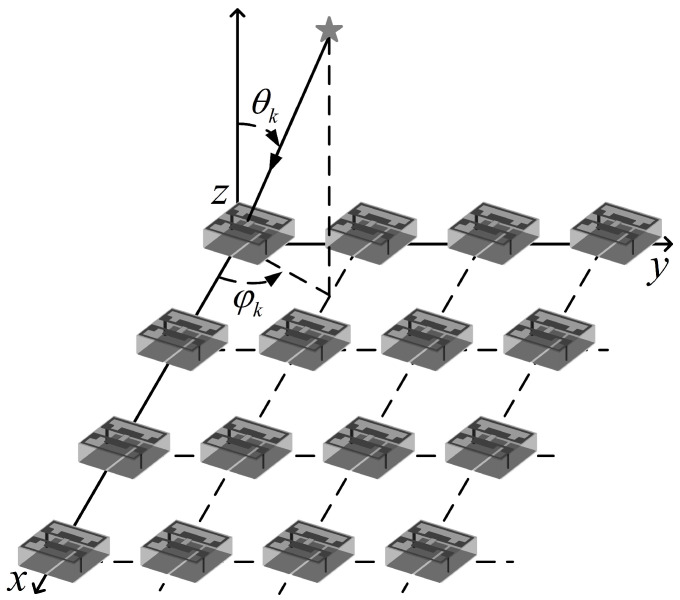
Signal arrives in RIS.

In order to construct the space–time asymmetry, the antenna is designed as in Figure 1 with M×M RIS elements. The model is used to estimate TD and DOA without designing signal source locations. Multipath wireless propagation channels are usually modeled as complex low-pass equivalent impulse responses. Following the channel estimation, the channel impact response of the RIS element at location (x, y) in the sth time interval is denoted by

(1) $$h_{x,y}^{\left(s\right)}\left(t\right)=\sum_{k=1}^{K}\alpha_{k}^{\left(s\right)}e^{j\beta_{k}^{\left(s\right)}}\delta \left(t-\tau_{k}\right)*\left[\delta \left(t-\xi_{k,x,y}\right)\Gamma \left(t\right)\right],$$

where *K* indicates the number of multipaths; $\alpha_{k}^{\left(s\right)}e^{j\beta_{k}^{\left(S\right)}}$ is the complex attenuation of the kth path; $\alpha_{k}^{\left(s\right)}$ is the amplitude; $\beta_{k}^{\left(s\right)}$ is the phase that is consistent with the uniform distribution of the density function U(0,2π).

Suppose the RIS element at the origin of the coordinate axis is the reference RIS element, and the propagation delay of the source reaching the reference RIS element through the kth path is $\tau_{k}$ in the multipath environment. The relative delay of the RIS element at location (x,y) with regard to the reference position is $\xi_{k,x,y}$, which is indicated by

(2) $$\xi_{k,x,y}=\frac{\lambda \sin\theta_{k}\left(x\cos\varphi_{k}+y\sin\varphi_{k}\right)}{2c},$$

in which λ is the impinging signal wavelength; $\left(\varphi_{k},\theta_{k}\right)$ is the direction of the incoming wave; Γ(t) is the coding coefficient of RIS, and we set its cycle to *T* with the ODFM symbol period. Furthermore, we have

(3) $$\Gamma \left(t\right)=\sum_{w=0}^{W-1}\Gamma_{w}g(t-m\psi ),\;(0\leq t\leq T)\;,$$

where the period *T* is uniformly divided into *W*; the coding coefficient of the wth time segment is a constant $\Gamma_{w}$; g(t) is a periodic unit pulse signal with a width of ψ=T/W, which can be expressed as

(4) $$g(t)\;=\;\left\{\begin{array}{cc} \; & 1,\;(0\leq t\leq T)\\ \; & 0,\;(0\leq t\leq T) \end{array}\right.,$$

which can be expressed in terms of the Fourier series as

(5) $$g\left(t\right)=\sum_{l=0}^{L-1}c_{l}e^{j2\pi \Delta flt},$$

where Δf=1/T is the OFDM subcarrier spacing, and *L* is the quantity of OFDM subcarriers. Moreover, $c_{l}$ is the Fourier series coefficient, which can be expressed as

(6) $$c_{l}=\frac{1}{W}\frac{\sin(\frac{l\pi }{W})}{\frac{l\pi }{W}}e^{-j\frac{l\pi }{W}}=\frac{1}{W}Sa\left(\frac{l\pi }{W}\right)e^{-j\frac{l\pi }{W}}.$$

We take (5) into (3) to obtain

(7) $$\begin{array}{cc} \Gamma (t) & =\sum_{l=0}^{L}\left[c_{l}\sum_{w=0}^{W-1}\Gamma_{w}e^{-jl_{g}\frac{2w\pi }{W}}\right]e^{j2\pi \Delta flt}\\ \; & =\sum_{l=0}^{L}\alpha_{\Gamma }e^{j\beta_{\Gamma }}e^{j2\pi \Delta flt} \end{array},$$

where $\alpha_{\Gamma }e^{j\beta_{\Gamma }}$ is the composite attenuation caused by RIS; $\alpha_{\Gamma }$ is the amplitude, and $e^{j\beta_{\Gamma }}$ is the phase, whose effects can be neglected by properly setting *W* and $\Gamma_{w}$ to achieve $\alpha_{\Gamma }e^{j\beta_{\Gamma }}\alpha e^{j\beta }\approx \alpha e^{j\beta }$.

### 2.2. Frequency-Domain Model

Performing the Fourier transform to (1), we obtain the Coded-Response of the lth subcarrier at the (x,y) RIS element as

(8) $$\begin{array}{cc} \;\;\;\;H_{l,x,y}^{\left(s\right)} & =\sum_{k=1}^{K}\alpha_{k}^{\left(s\right)}e^{j\beta_{k}^{\left(s\right)}}e^{-j2\pi \left(f_{c}+\Delta fl\right)\tau_{k}}\\ \; & (e^{-j2\pi \left(f_{c}+\Delta fl\right)\xi_{k,x,y}}*\Gamma \left(f\right))+n_{l,x,y}^{\left(s\right)}\\ \; & \;\;\;=\;\;\;\sum_{k=1}^{K}\alpha_{k}^{\left(s\right)}e^{j\beta_{k}^{\left(s\right)}}e^{-j2\pi \left(f_{c}+\Delta fl\right)\tau_{k}}e^{-j2\pi f_{c}\xi_{k,x,y}}+n_{l,x,y}^{\left(s\right)} \end{array},$$

where $f_{c}$ is the carrier frequency, and $n_{l,x,y}^{\left(s\right)}$ is additive white Gaussian noise with power $\sigma^{2}$. According to (8), the Coded-Response of lth subcarrier is given by

(9) $$\begin{array}{cc} H_{l}^{\left(s\right)} & =\sum_{k=1}^{K}\left[\begin{array}{c} H_{l,0,0}^{\left(s\right)}\\ H_{l,0,1}^{\left(s\right)}\\ \vdots \\ H_{l,M-1,M-1}^{\left(s\right)} \end{array}\right]\\ \; & =\sum_{k=1}^{K}A_{\tau l}\left(\tau_{k}\right)⊙A_{\xi }\left(\xi_{k}\right)\rho_{k}^{\left(s\right)}+n_{l}^{\left(s\right)} \end{array},$$

where

(10) $$A_{\tau_{l}}\left(\tau_{k}\right)=e^{-j2\pi \left(f_{c}+\Delta fl\right)\tau_{k}},$$

(11) $$A_{\xi }\left(\xi_{k}\right)={\left[\begin{array}{ccc} e^{-j2\pi f_{c}\xi_{k,0,0}} & \cdots & e^{-j2\pi f_{c}\xi_{k,M-1,M-1}} \end{array}\right]}^{T},$$

(12) $$\rho_{k}^{\left(s\right)}=\alpha_{k}^{\left(s\right)}e^{j\beta_{k}^{\left(s\right)}},$$

(13) $$n_{l}^{\left(s\right)}={\left[\begin{array}{cccc} n_{l,0,0}^{\left(s\right)} & n_{l,0,1}^{\left(s\right)} & \cdots & n_{l,M-1,M-1}^{\left(s\right)} \end{array}\right]}^{T}.$$

The subcarriers in the OFDM signal can be similar to the RIS arrays based on space–time equivalence. Based on the signal subspace algorithms, a space–frequency Coded-Response matrix can be constructed with the expression

(14) $$\begin{array}{cc} \; & H^{\left(s\right)}=\sum_{k=1}^{K}\left[\begin{array}{c} A_{\tau 0}\left(\tau_{k}\right)\\ A_{\tau 1}\left(\tau_{k}\right)\\ \vdots \\ A_{\tau (L-1)}\left(\tau_{k}\right) \end{array}\right]⊙A_{\xi }\left(\xi_{k}\right)\rho_{k}^{\left(s\right)}+n^{\left(s\right)}\\ \; & =\sum_{k=1}^{K}A_{\tau }\left(\tau_{k}\right)⊙A_{\xi }\left(\xi_{k}\right)\rho_{k}^{\left(s\right)}+n^{\left(s\right)} \end{array},$$

where

(15) $$n^{\left(s\right)}={\left(\begin{array}{cccc} {n^{\left(s\right)}}_{0}^{T} & {n^{\left(s\right)}}_{1}^{T} & \cdots & {n^{\left(s\right)}}_{L-1}^{T} \end{array}\right)}^{T}.$$

In addition, if there are an overall *S* time intervals, then (14) is represented as

(16) $$H=A_{\tau }\left(\tau \right)⊙A_{\xi }\left(\xi \right)\rho +n,$$

where

(17) $$H=\left[\begin{array}{cccc} H^{\left(1\right)} & H^{\left(2\right)} & \cdots & H^{\left(S\right)} \end{array}\right],$$

(18) $$A_{\tau }\left(\tau \right)=\left[\begin{array}{cccc} A_{\tau }\left(\tau_{1}\right) & A_{\tau }\left(\tau_{2}\right) & \cdots & A_{\tau }\left(\tau_{K}\right) \end{array}\right],$$

(19) $$A_{\xi }\left(\xi \right)=\left[\begin{array}{cccc} A_{\xi }\left(\xi_{1}\right) & A_{\xi }\left(\xi_{2}\right) & \cdots & A_{\xi }\left(\xi_{K}\right) \end{array}\right],$$

(20) $$\rho ={\left[\begin{array}{cccc} \rho_{1} & \rho_{1} & \cdots & \rho_{K} \end{array}\right]}^{T},$$

(21) $$\rho_{k}=\left[\begin{array}{cccc} \rho_{k}^{\left(1\right)} & \rho_{k}^{\left(2\right)} & \cdots & \rho_{k}^{\left(S\right)} \end{array}\right],$$

(22) $$n=\left[\begin{array}{cccc} n^{\left(1\right)} & n^{\left(2\right)} & \cdots & n^{\left(S\right)} \end{array}\right].$$

The time-domain encoding of RIS transforms the combination of $A_{\tau }\left(\tau \right)$ and $A_{\xi }\left(\xi \right)$ from Hadmard to Khatri–Rao, providing the possibility of solving TD and DOA separately. The coded signal model converts the time-domain structure into a frequency-domain structure so that it is still suitable for the time-domain method. Additionally, there are two clear benefits to building a Coded-Response for joint TD and DOA estimation. On the one hand, the simultaneous acquisition of TD and DOA parameters enables single-station localization to reduce the localization system overhead. On the other hand, the proposed joint estimation approach leads to an increase in *L* times aperture and $M^{2}$ times bandwidth compared to reality, which is better than the individual estimation performance.

## 3. The Joint TD and DOA Estimation

### 3.1. Tensor Approach

In this section, we represent the Coded-Response (16) into a tensor. The tensorial form can reveal signal model structure clearly, which enables us to realize signal and noise subspaces more accurately. In addition, the proposed method improves the robustness in a coherent multipath environment with a tensor form of

(23) $$H=A_{\tau }\left(\tau \right)\circ A_{\xi }\left(\xi \right)\circ \rho^{T}+N,$$

where $H\in C^{M^{2}\times L\times S}$, and N is additive white Gaussian noise in the same dimension as H with power of $\sigma^{2}$. In (23), the tensor covariance matrix $R{}_{\;H\;}\;\in \;{\;C\;}^{M^{2}\times L\times M^{2}\times L}$ can be obtained as

(24) $$\begin{array}{cc} \; & R{}_{H}=E\left[<H,H^{*}{>}_{\left\{3\right\}}\right]\\ \; & =\sum_{k=1}^{K}\beta_{k}^{2}A_{\tau }\left(\tau_{k}\right)\circ A_{\xi }\left(\xi_{k}\right)\circ A_{\tau }^{*}\left(\tau_{k}\right)\circ A_{\xi }^{*}\left(\xi_{k}\right)+N_{R} \end{array}\prime$$

where $\beta_{k}^{2}=E\left[\rho_{k}^{T}{\left(\rho_{k}^{T}\right)}^{*}\right]$ is the complex decay of the kth path, and $N_{R}=E\left[{\langle N,N^{*}\rangle }_{\left\{3\right\}}\right]$ is the noise term. In practice, the tensor covariance matrix $R{}_{H}$ can be estimated as

(25) $${\hat{R}}_{H}=\frac{1}{S}{\langle H,H^{*}\rangle }_{\left\{3\right\}}.$$

The CANDECOMP/PARAFAC decomposition (CPD) [26] is a common method for splitting a high-dimensional tensor. We perform CPD on the four-dimensional tensor ${\hat{R}}_{H}$ to obtain a sum $R_{CP}\in C^{M^{2}\times K,L\times K,M^{2}\times K,L\times K}$ of component rank-one tensors as

(26) $$R_{CP}=\left[T_{CP}\right],\left[A_{CP}\right],\left[T_{CP}^{*}\right],\left[A_{CP}^{*}\right].$$

Due to the milder uniqueness condition of tensor decomposition and the asymmetry of the constituent factor vectors in (23), there is

(27) $$\begin{array}{cc} \; & \mathrm{rank}\left({T\;}_{CP}\;\right)\;+\;\mathrm{rank}\left(\;A_{CP}\;\right)\;+\;\mathrm{rank}\left(T{\;}_{CP}{\;}^{*}\;\right)\;+\;\mathrm{rank}\left(\;A_{CP}{\;}^{*}\;\right)\;\;\geq \;\;2K\;\;+\;\;N\;\;-\;\;1\\ \; & \Rightarrow K+K+K+K\geq 2K+N-1\\ \; & \Rightarrow 2K\geq 3 \end{array}.$$

*N* is the amount of matrices obtained by CPD or the dimension of the decomposed matrix, which is four in this algorithm. Therefore, when the number of sources is not unique, the proposed algorithm satisfies the uniqueness of CPD [26]. Further, we extract the signal subspace $T_{CP}$ from $R_{CP}$ for TD estimation and the signal subspace $A_{CP}$ for DOA estimation.

### 3.2. TD Estimation

Since the phases of TD and DOA are asymmetric in frequency, it allows TD to be estimated separately while suppressing the coherent signal. To demonstrate this process, we will fold the $M^{2}L\times 1$ received vector (14) into an $L\times M^{2}$ matrix as follows

(28) $$H_{Tfold}^{\left(s\right)}\left(p,q\right)=H^{\left(s\right)}\left(M^{2}\left(p-1\right)+q\right),$$

where $H_{Tfold}^{\left(s\right)}\in C^{L\times M^{2}}$ is a new observation matrix of p=1,2,…,L and $q=1,2,\ldots ,M^{2}$. Thus, TD and DOA can be decoupled in phase as

(29) $$H_{Tfold}^{\left(s\right)}=\sum_{k=1}^{K}A_{\tau }\left(\tau_{k}\right)A_{\xi }^{T}\left(\xi_{k}\right)\rho_{k}^{\left(s\right)}+n_{{Tfold}^{\prime }}^{\left(s\right)}$$

where $n_{Tfold}^{\left(s\right)}$ denotes the noise term and still follows the white Gaussian distribution. Further, $H_{Tfold}^{\left(s\right)}$ can be considered as a sample collected by the RIS at sth time interval. Here, we define

(30) $$F^{\left(s\right)}={\left[\begin{array}{cccc} A_{\xi }^{T}\left(\xi_{1}\right)\rho_{1}^{\left(s\right)} & A_{\xi }^{T}\left(\xi_{2}\right)\rho_{2}^{\left(s\right)} & \cdots & A_{\xi }^{T}\left(\xi_{K}\right)\rho_{K}^{\left(s\right)} \end{array}\right]}^{T}.$$

Then the covariance matrix corresponding to (29) is

(31) $$\begin{array}{cc} R_{Tfold} & =\frac{1}{S}\sum_{s=1}^{S}H_{Tfold}^{\left(s\right)}{\left(H_{Tfold}^{\left(s\right)}\right)}^{H}\\ \; & =A_{\tau }\left(\tau \right)R_{F}A{\left(\tau \right)}^{H}+\sigma^{2}I_{L} \end{array},$$

where

(32) $$\begin{array}{cc} \; & R_{F}=\frac{1}{S}\sum_{s=1}^{S}F^{\left(s\right)}{\left(F^{\left(s\right)}\right)}^{H}\\ \; & =R_{\xi }⊕R_{\rho } \end{array},$$

where

(33) $$R_{\xi }=\left[\begin{array}{ccc} A_{\xi }^{T}\left(\xi_{1}\right)A_{\xi }^{*}\left(\xi_{1}\right) & \cdots & A_{\xi }^{T}\left(\xi_{1}\right)A_{\xi }^{*}\left(\xi_{K}\right)\\ \vdots & \ddots & \vdots \\ A_{\xi }^{T}\left(\xi_{K}\right)A_{\xi }^{*}\left(\xi_{1}\right) & \cdots & A_{\xi }^{T}\left(\xi_{K}\right)A_{\xi }^{*}\left(\xi_{K}\right) \end{array}\right],$$

(34) $$R_{\rho }=\frac{1}{S}\sum_{s=1}^{S}\rho^{\left(s\right)}{\left(\rho^{\left(s\right)}\right)}^{H}.$$

Note that $A_{\xi }^{T}\left(\xi_{k}\right)A_{\xi }^{*}\left(\xi_{i}\right)$, k,i=1,2,⋯,K can only achieve maximum value if k=i. Since the relative delays of the signals are different from each other, $R_{\xi }$ is a diagonally dominant matrix which reduces the singularity of the signal correlation matrix when the signals are correlated. Combining with (32), it can be seen that in the coherent environment even if $\mathrm{rank}(R_{\rho })<K$ there is still $\mathrm{rank}(R_{\xi })<K$ enabling $\mathrm{rank}(R_{F})<K$. Therefore, if the proposed method can obtain the signal or noise subspace of $A_{\tau }\left(\tau \right)$ alone for TD estimation, the method is adaptive to coherent signals.

Then we obtain the corresponding signal subspace $T_{CP}$ of $A_{\tau }\left(\tau \right)$ from (26) and construct the corresponding noise subspace $U_{nt}U_{nt}^{H}$ as follows

(35) $$U_{nt}U_{nt}^{H}=I-\mathrm{orth}\left(T_{CP}\right)\mathrm{orth}{\left(T_{CP}\right)}^{H}.$$

Then the spatial spectrum expression of TD estimation is

(36) $$P\left(\tau \right)=\frac{1}{T{\left(\tau \right)}^{H}\left(U_{nt}U_{nt}^{H}\right)T\left(\tau \right)}.$$

We can use the one-dimensional (1D) MUSIC algorithm to solve the value of $\hat{\tau }$, which has a higher estimation accuracy and significantly reduces the complexity compared to the three-dimensional (3D) MUSIC algorithm. Further, in the proposed tensor model, the separate TD estimates are not affected by the coherent multipath environment.

### 3.3. DOA Estimation

As the phases of TD and DOA are asymmetric in frequency, it allows DOA to be estimated separately while suppressing the coherent signal. To demonstrate this process, we will fold the $M^{2}L\times 1$ received vector (14) into an $M^{2}\times L$ matrix as follows

(37) $$H_{Afold}^{\left(s\right)}\left(p,q\right)=H^{\left(s\right)}\left(p+M^{2}\left(q-1\right)\right),$$

where $H_{Afold}^{\left(s\right)}\in C^{M^{2}\times L}$ is a new observation matrix of $p=1,2,\ldots ,M^{2}$ and q=1,2,…,L. Thus, DOA and TD can be decoupled in phase as

(38) $$H_{Afold}^{\left(s\right)}=\sum_{k=1}^{K}A_{\xi }\left(\xi_{k}\right)A_{\tau }^{T}\left(\tau_{k}\right)\rho_{k}^{\left(s\right)}+n_{{Afold}^{\prime }}^{\left(s\right)}$$

where $n_{Afold}^{\left(s\right)}\in C^{M^{2}\times L}$ stands for the complex white Gaussian noise. The remaining proof process is similar to (31)–(34), and the proposed method is adaptive to coherent signals if the signal or noise subspace of $A_{\xi }\left(\xi \right)$ can be obtained separately for DOA estimation. Then we obtain the corresponding signal subspace $A_{CP}$ of $A_{\xi }\left(\xi \right)$ from (26) and construct the corresponding noise subspace $U_{na}U_{na}^{H}$ as follows

(39) $$U_{na}U_{na}^{H}=I-\mathrm{orth}\left(A_{CP}\right)\mathrm{orth}{\left(A_{CP}\right)}^{H}.$$

Then the spatial spectrum expression of the DOA estimate can be obtained as

(40) $$P\left(\theta ,\varphi \right)=\frac{1}{A{\left(\theta ,\varphi \right)}^{H}\left(U_{na}U_{na}^{H}\right)A\left(\theta ,\varphi \right)}.$$

We can use the two-dimensional (2D) MUSIC algorithm to solve the values of $\left(\hat{\theta },\hat{\varphi }\right)$, which has a higher estimation accuracy and greatly reduces the complexity compared to the 3D MUSIC algorithm. Further, in the proposed tensor model, the separate TD estimates are not affected by the coherent multipath environment.

### 3.4. Algorithm Steps

The major steps of the proposed algorithm are presented in Algorithm 1.

**Algorithm 1** Algorithm steps.
**step1:**
The Coded-Response H in tensor form is constructed according to (23).
**step2:**
The tensor covariance matrix $\overset{⌢}{R}{}_{H}$ is constructed according to (25).
**step3:**
Perform a CPD of ${\hat{R}}_{H}$, which solves the signal subspaces $T_{CP}$ and $V_{CP}$, and then the corresponding noise subspaces and are obtained by (35) and (39), respectively. $U_{nt}U_{nt}^{H}$ and $U_{na}U_{na}^{H}$.
**step4:**
Proceed to conduct a 1D spectral peak search for $U_{nt}U_{nt}^{H}$ to solve the $\hat{\tau }$ by (36), and a 2D spectral peak search for $U_{na}U_{na}^{H}$ to solve the $\left(\hat{\theta },\hat{\varphi }\right)$ by (40).

## 4. Algorithms Complexity

In this part, we analyze the complexity of the proposed algorithm under the Coded-Response model and compare it with the 3D spectral peak search method (3D-MUSIC). To analyze the accuracy in the next section, we also compare the complexity of the algorithm (joint-smooth) employing smoothing preprocessing [22], which reduces the dimensions of the joint estimation model.

**Figure 2:**
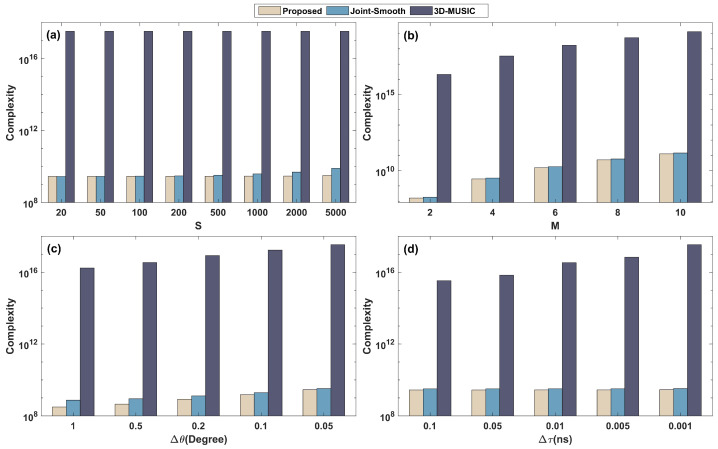
Comparison of complexity: (**a**) with *S* where M=4, $\Delta \theta \;\;\;=\;\;\;0.05^{\circ }$, Δτ=0.001ns; (**b**) with *M* where S=500, $\Delta \theta \;\;\;=\;\;\;0.05^{\circ }$, Δτ=0.001ns; (**c**) with Δθ where M=4, S=500, Δτ=0.001ns; (**d**) with Δτ where M=4, S=500, $\Delta \theta \;\;\;=\;\;\;0.05^{\circ }$.

The related complexity is mainly split into vector-based covariance matrix computation, tensor-based covariance matrix computation, eigenvalue decomposition, and CPD and 1D spectrum peak search, which are $O(SM^{4}L^{2})$, $O(SM^{2}L^{2})$, $O(M^{3})$, $O(2^{N}KM^{4}L^{2}+NK^{3})$, and O(M(M−K)G), separately, where *G* represents the quantity of spectral points in the 1D search. Hence, the proposed complexity is $O\left(M^{2}L^{2}\right)\left(S+2^{N}KM^{2}\right)+NK^{3}+M^{2}\left(M^{2}-K\right)G_{\varphi }G_{\theta }+L\left(L-K\right)G_{\tau }$. The cost of the 3D-MUSIC is $O(\left(S+M^{2}L\right)M^{4}L^{2}+M^{2}L\left(M^{2}L-K\right)G_{\varphi }G_{\theta }G_{\tau })$, as well as the smoothing preprocessing method (joint-smooth) is $O(SM^{4}L^{2}+M^{6}+L^{3}+M^{2}\left(M^{2}-K\right)G_{\varphi }G_{\theta }+L\left(L-K\right)G_{\tau })$, in which $G_{\varphi }$, $G_{\theta }$, and $G_{\tau }$ denote the quantity of searches for azimuth, elevation, and propagation delay, separately. To make the comparison clear, Table 2 summarizes the complexity of all methods. Additionally, we have compared the complexity of the algorithms in terms of time intervals (S), the quantity of RIS elements (M), and the searching step (Δτ, Δφ=Δθ, in which $G_{\varphi }=360/\Delta \varphi$, $G_{\theta }=90/\Delta \theta$) in Figure 2a–d, separately.

From Figure 2, the 3D-MUSIC complexity is substantial because of the large quantity of spectral points, specifically in the small spectral steps. The high complexity of the 3D-MUSIC algorithm brings a very high estimation accuracy, but it cannot be used for coherent signals. In contrast, the proposed and joint-smooth methods are used to reduce the spectral peak search dimension by tensor CPD and full-dimensional smoothing preprocessing, which leads to a significant reduction in complexity. Thus, both algorithms have similar complexity, and both are applicable in coherent environments. Due to the large-scale smoothing preprocessing that leads to more aperture loss, the estimation performance of the joint-smooth method is lower than that of the proposed method.

## 5. Simulation Results

Here, we present simulation results which focus on the analysis of the proposed algorithm with joint-smooth. In addition, the proposed algorithm is compared with 3D-MUSIC in the independent multipath environment.

### 5.1. Performance at Low SNR

**Figure 3:**
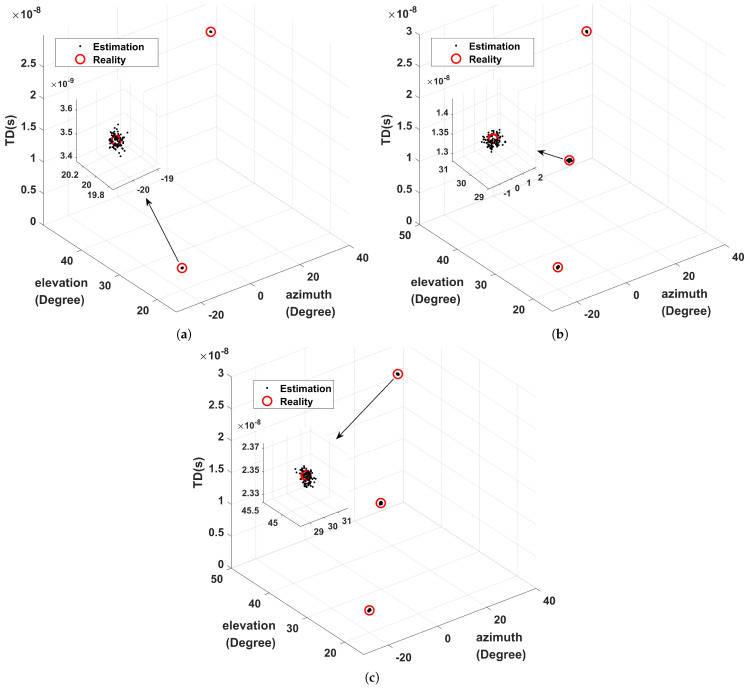
Estimated distribution at SNR=−5dB: (**a**) two coherent components; (**b**) two coherent components and one independent component; (**c**) three independent components.

Three cases are designed in this paper. First, suppose that the coherent signals are two, the corresponding delays are 3.5ns and 23.5ns, related azimuth angles are $-20^{\circ }$ and $30^{\circ }$, and corresponding elevation angles are $20^{\circ }$ and $45^{\circ }$, respectively. Secondly, we append an independent signal with 23.5ns time delay, $30^{\circ }$ azimuth angle, and $45^{\circ }$ elevation angle. Thirdly, we assume all three signals are independent. Moreover, Q=100 is set and the distribution of TD and DOA under S=500, and SNR=−5dB is determined, as illustrated in Figure 3. Figure 3 shows that this algorithm has the ability to estimate TD and DOA values in various situations. In addition, the proposed algorithm has good robustness and performance under low SNR.

### 5.2. Performance versus SNR

**Figure 4:**
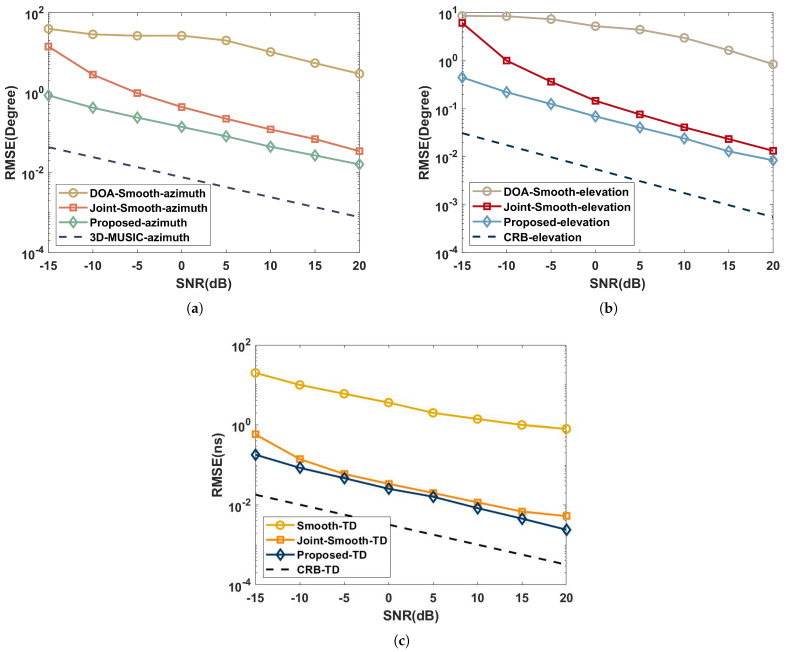
Performance comparison with SNR in a coherent multipath environment: (**a**) azimuth; (**b**) elevation; (**c**) time delay.

**Figure 5:**
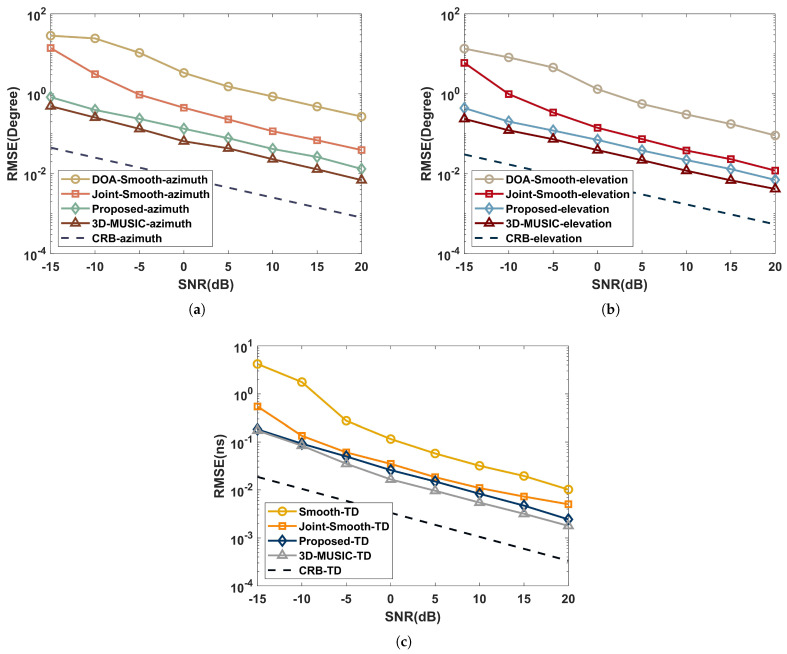
Performance comparison with SNR in an independent multipath environment: (**a**) azimuth; (**b**) elevation; (**c**) time delay.

This part analyzes the capabilities of the proposed algorithm, Joint-smooth, 3D-MUSIC, TD estimation [27], DOA estimation [28], and Cramer–Rao bound (Appendix A) in the coherent and independent multipath environments. Suppose the multipath signals are three with the same parameters as simulated in Section 5.1. We set Q=100, S=500, and the spectral steps of Δτ=0.001ns and $\Delta \varphi =\Delta \theta =0.05^{\circ }$. Further, the RMSE performance versus SNR with ranges of −15dB to 20dB in 5dB intervals is shown in Figure 4 and Figure 5.

As shown in Figure 4, the joint estimation algorithms (joint-smooth and proposed) in the coherent multipath environment work significantly better than the performance of the independent estimation algorithms (DOA-Smooth and Smooth-TD) due to the aperture and bandwidth enhancement brought by the joint approach. Combined with the complexity comparison of Section 4, it can be concluded that the proposed has a higher estimation accuracy than the joint-smooth, while the complexity is very close for $O\left(2.88\times 10^{9}\right)$ and $O\left(3.32\times 10^{9}\right)$. The the proposed avoids the aperture loss in processing coherent signals by the time-domain coding function of the RIS.

As illustrated in Figure 5, the RMSE of 3D-MUSIC is closer to CRB than the proposed in the independent multipath environment because the CPD process of the proposed loses part of the extended structure of the subspace. However, the complexity of 3D-MUSIC is $O\left(3.39\times 10^{17}\right)$, which is much higher than the proposed and difficult to accept in practical scenarios.

### 5.3. Performance versus Time Intervals

To highlight the impact of time intervals on RMSE, we set SNR=15dB, the quantity of time intervals varies at the range of S={20,50,100,200,500,1000,2000,5000}, and the rest of the simulation parameters are identical to those simulated in Section 5.2.

**Figure 6:**
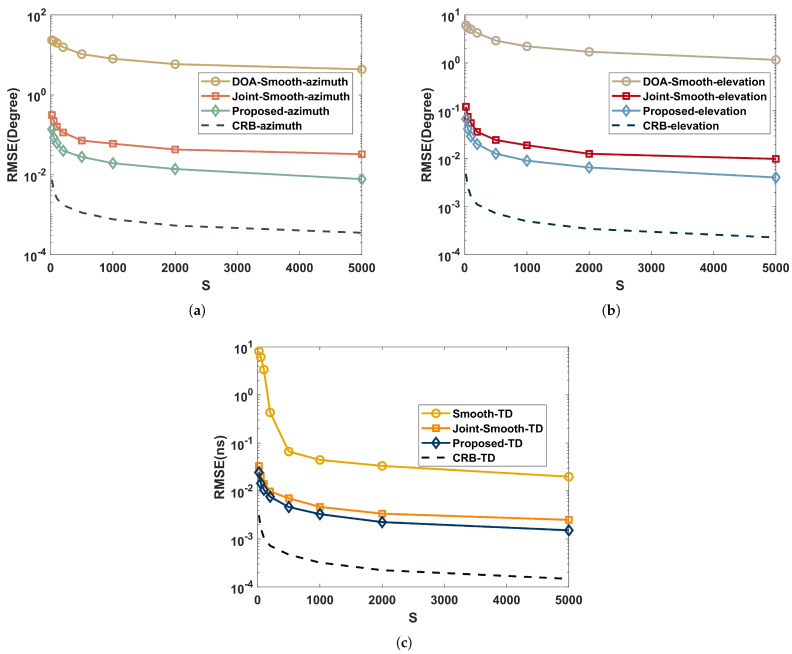
Performance comparison with time intervals in a coherent multipath environment: (**a**) azimuth; (**b**) elevation; (**c**) time delay.

As shown in Figure 6, when processing coherent signals, the joint-smooth and the proposed have significantly better performance than DOA-Smooth and Smooth-TD due to the aperture and bandwidth improvement brought by the joint approach. Further, the proposed avoids the aperture loss of the current smoothing method when processing coherent signals by the time-domain coding function of RIS. Therefore, its estimation performance is better than the joint-smooth.

**Figure 7:**
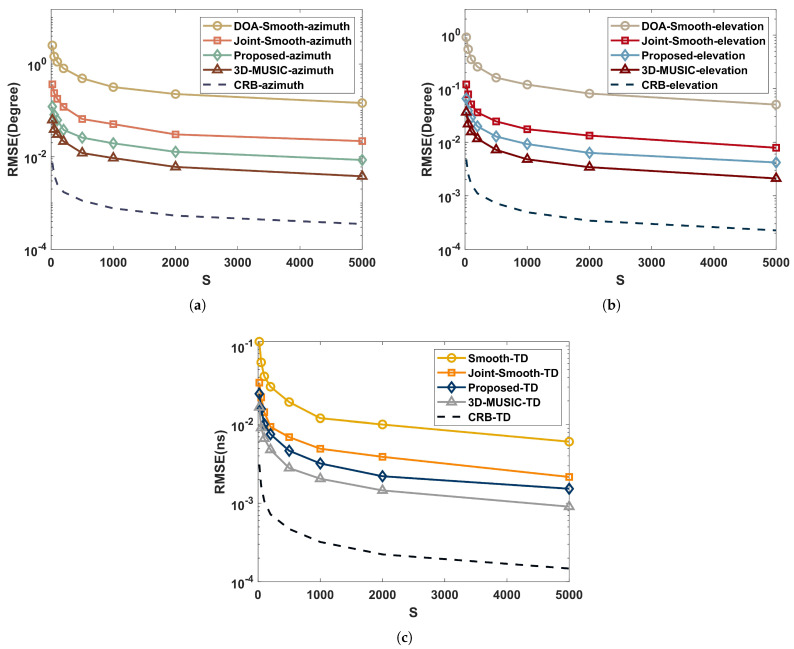
Performance comparison with time intervals in an independent multipath environment: (**a**) azimuth; (**b**) elevation; (**c**) time delay.

As shown in Figure 7, in the independent multipath environment, 3D-MUSIC has a lower RMSE than the proposed, which is due to the fact that the CPD process of the proposed loses part of the subspace expansion structure. Other conclusions are the same as in Figure 6.

In summary, the RMSE of the proposed algorithm reduces as the time interval increases, although the reduction gradually achieves a plateau. Additionally, more algorithms are adapted to independent environments, and the estimated performance is typically better.

### 5.4. Performance versus RIS Elements

For highlighting the effect of RIS elements on RMSE, we set SNR=15dB, the number of RIS elements varies in the range of M={3,4,5,6,7,8,9,10}, and the rest of the simulation parameters are the same as those simulated in Section 5.2.

**Figure 8:**
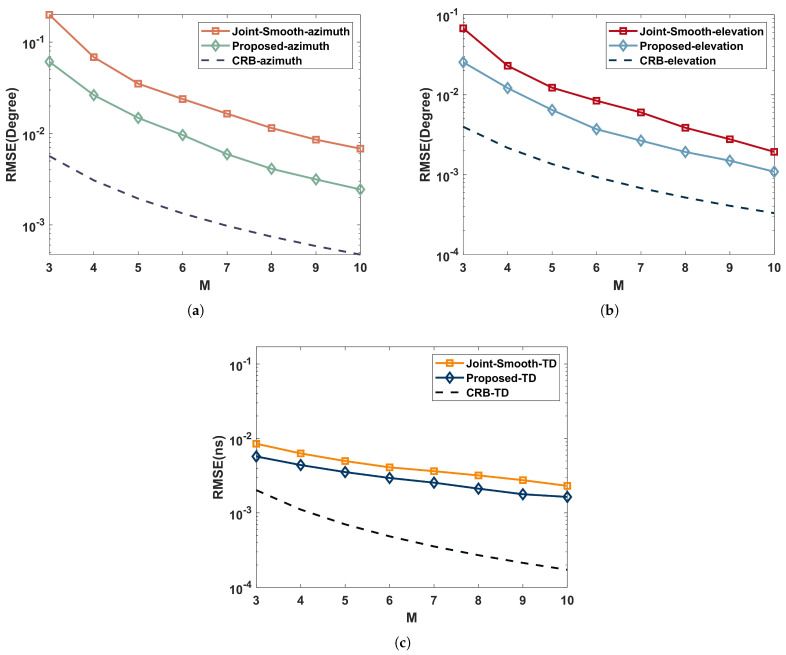
Performance comparison with RIS elements in a coherent multipath environment: (**a**) azimuth; (**b**) elevation; (**c**) time delay.

In Figure 8, we simulate the performance of the joint estimation algorithm in a coherent environment. As illustrated in (a) and (b), the performance of the joint estimation DOA gets better as the number of RIS elements increases. However, the trend flattens out, which is because the algorithm performance is mainly limited by the SNR when there are enough RIS elements.

As shown in (c), the performance of the joint estimation TD also gets better as the number of RIS elements increases. However, it is much less affected due to the fact that TD estimation is mainly limited by the signal bandwidth. Even though the increase of RIS elements in the joint estimation model expands the signal bandwidth, its effect is not infinitely increasing.

## 6. Conclusions

For the joint estimation for TD and DOA of coherent signals on a single station system, we propose an algorithm based on RIS and OFDM techniques in this paper. The proposed algorithmic model integrates the RIS array response and the channel frequency response of OFDM subcarriers in tensor form to extend the bandwidth and aperture and achieve better estimation. The appropriate combination of the RIS time-domain coding function and the tensor structure allows the algorithm to estimate coherent signals with reduced complexity. Compared with the current algorithm, the proposed algorithm has better estimation performance in processing coherent signals without the aperture loss of the smoothing process and can rely on the advantage of joint estimation for single station localization systems. In this paper, we begin by presenting the signal model and the algorithmic procedure, which has detailed derivations and proofs. Then, to reflect the fast estimation of the proposed algorithm, we compare the related algorithms in terms of complexity. Finally, the validity and robustness of the algorithm are verified by simulation experiments. In comparison with current algorithms, the proposed algorithm provides more effective parameter estimation with lower complexity in coherent or mixed multipath environments.

## Figures and Tables

**Table 1:** Notations.

Transpose	${\left(•\right)}^{T}$
Conjugate	${\left(•\right)}^{*}$
Hermitian transpose	${\left(•\right)}^{H}$
Khatri-Rao product	⊙
Kronecker product	⊗
Hadamard product	⊕
Tensor outer product	∘
Convolution	∗
Identity matrix	***I***
Statistical expectation	E{•}
Orthogonalization	$\mathrm{orth}\left[•\right]$
Tensor contraction along the qth dimension	${\langle •,•\rangle }_{\left\{q\right\}}$

**Table 2:** Comparison of complexity.

Algorithm	Complexity
Proposed	$O\left(M^{2}L^{2}\right)\left(S+2^{N}KM^{2}\right)+NK^{3}+L\left(L-K\right)G_{\tau }+M^{2}\left(M^{2}-K\right)G_{\varphi }G_{\theta }$
3D-Music	$O(\left(S+M^{2}L\right)M^{4}L^{2}+M^{2}L\left(M^{2}L-K\right)G_{\varphi }G_{\theta }G_{\tau })$
Joint-Smooth	$O(SM^{4}L^{2}+M^{6}+L^{3}+M^{2}\left(M^{2}-K\right)G_{\varphi }G_{\theta }+L\left(L-K\right)G_{\tau })$

## Data Availability

The data that support the findings of this study are available upon request from the authors.

## Appendix A. Proof of Proposition

Definition Cramer–Rao bound: The CRB [29] is a threshold for the unbiased estimation variance of the proposed model and can be used as a performance reference benchmark.

Firstly, we assume that the parameter estimates are all performed individually with *S* time intervals, so the joint probability density function is

(A1) $$\begin{array}{cc} \; & f\left(H\left(1\right),\cdots ,H\left(S\right)\right)=\\ \; & \;\;\frac{1}{{\left(2\pi \right)}^{M^{2}LS}{\left(\sigma^{2}/2\right)}^{M^{2}LS}}e^{-\frac{1}{\sigma^{2}}\sum_{s=1}^{S}{\left(H\left(s\right)-A\rho \left(s\right)\right)}^{H}\left(H\left(s\right)-A\rho \left(s\right)\right)} \end{array}.$$

Then taking the log-likelihood function of (A1), we obtain

(A2) $$\begin{array}{cc} \; & \;\;\;\;\;Lo\left(H\left(1\right),\;\cdots \;,H\left(S\right)\right)\;=\;-M^{2}LS\ln\left(2\pi \right)-M^{2}LS\ln\left(\sigma^{2}/2\right)\\ \; & -\frac{1}{\sigma^{2}}\sum_{s=1}^{S}{\left(H\left(s\right)-A\rho \left(s\right)\right)}^{H}\left(H\left(s\right)-A\rho \left(s\right)\right) \end{array}.$$

Define $\eta ={\left[\emptyset^{T},\theta^{T},\varphi^{T}\right]}^{T}$. In addition, $\tilde{\rho }\left(s\right)$ and $\bar{\rho }\left(s\right)$ are the imaginary part and real part of ρ(s), respectively, which are indicated as ρ¯(s)=Reρ(s) and ρ˜(s)=Imρ(s). The Fisher information matrix is $\Omega =\left[E\left(\psi \psi^{T}\right)\right]$, where

(A3) $$\psi^{T}\;=\partial Lo/\partial \left[\begin{array}{ccccccc} \sigma^{2}\; & \;{\bar{\rho }}^{T}\left(1\right)\; & \;{\tilde{\rho }}^{T}\left(1\right)\; & \cdots & \;{\bar{\rho }}^{T}\left(S\right)\; & \;{\tilde{\rho }}^{T}\left(S\right)\; & \;\eta^{T} \end{array}\right].$$

For the Fisher information matrix, the CRB of η conforms

(A4) CRBη=σ22∑s=1SReFHsBHPA⊥BFs−1,

where $P_{A}^{⊥}=I-P_{A}^{}=I-A{\left(A^{H}A\right)}^{-1}A^{H}$, $F\left(s\right)=I⊗diag\left(\rho \left(s\right)\right)$

We perform simulations using OFDM signals with L=64 subcarriers, fast Fourier transform period $T_{\mathrm{FFT}}=32\;\mathrm{us}$, carrier frequency $f_{c}=2.4\;\mathrm{GHz}$, and bandwidth B=20MHz. Used UPA includes 4×4 RIS elements. We set the spectrum steps of Δτ=0.001ns and $\Delta \theta =0.05^{\circ }$. For evaluating the precision of the methods, we calculate the root mean square error (RMSE) by

(A5) $$\mathrm{RMSE}=\sqrt{\frac{1}{QK}\sum_{q=1}^{Q}{∥\lambda -{\hat{\lambda }}_{i}∥}^{2}},$$

in which *Q*, ${\hat{\lambda }}_{i}$, and λ are the amount of Monte Carlo simulations, the ith estimated values and the true values, respectively.

## References

[1] Zhang L., Chen X.Q., Liu S., Zhang Q., Zhao J., Dai J.Y., Bai G.D., Wan X., Cheng Q., Castaldi G. (2018). Space-time-coding digital metasurfaces. Nat. Commun..

[2] Zhang L., Chen M.Z., Tang W., Dai J.Y., Miao L., Zhou X.Y., Jin S., Cheng Q., Cui T.J. (2021). A wireless communication scheme based on space-and frequency-division multiplexing using digital metasurfaces. Nat. Electron..

[3] He J., Wymeersch H., Sanguanpuak T., Silvén O., Juntti M. Adaptive beamforming design for mmwave ris-aided joint localization and communication. Proceedings of the 2020 IEEE Wireless Communications and Networking Conference Workshops (WCNCW).

[4] Chen P., Yang Z., Chen Z., Guo Z. (2021). Reconfigurable intelligent surface aided sparse doa estimation method with non-ula. IEEE Signal Process. Lett..

[5] Jin E.-S., Park H. A novel frequency domain preamble detection for ofdm based wlan systems. Proceedings of the 2020 International Conference on Information and Communication Technology Convergence (ICTC).

[6] Kumar T.A., Anjaneyulu L. Channel estimation techniques for multicarrier ofdm 5g wireless communication systems. Proceedings of the 2020 IEEE 10th International Conference on System Engineering and Technology (ICSET).

[7] Guo C.-L., Wei L., Qi W.-D. (2016). Channel estimation aided accurate toa estimation for ofdm systems in nlos environments. Wireless Communication and Sensor Network, Proceedings of the International Conference on Wireless Communication and Sensor Network (WCSN 2015), Changsha, China, 12–13 December 2015.

[8] Pradhan C., Li A., Song L., Li J., Vucetic B., Li Y. (2020). Reconfigurable intelligent surface (ris)-enhanced two-way ofdm communications. IEEE Trans. Veh. Technol..

[9] He Z., Shen H., Xu W., Zhao C. (2022). Low-cost passive beamforming for ris-aided wideband ofdm systems. IEEE Wirel. Commun. Lett..

[10] Jeong S., Farhang A., Perović N.S., Flanagan M.F. (2021). Low-complexity joint cfo and channel estimation for ris-aided ofdm systems. IEEE Wirel. Commun. Lett..

[11] Zheng Y., Sheng M., Liu J., Li J. (2019). Exploiting aoa estimation accuracy for indoor localization: A weighted aoa-based approach. IEEE Wirel. Commun. Lett..

[12] Kim S., Kim B., Lee J. (2020). Low-complexity-based rd-music with extrapolation for joint toa and doa at automotive fmcw radar systems. J. Sens..

[13] Cao F., Li M. Frequency domain doa estimation and tracking of uwb signals. Proceedings of the 2010 6th International Conference on Wireless Communications Networking and Mobile Computing (WiCOM).

[14] Ni H., Ren G., Chang Y. (2009). Novel toa estimation algorithm for ofdm wireless networks. J. Xidian Univ..

[15] Jiang H., Cao F., Ding R. Propagator method-based toa estimation for uwb indoor environment in the presence of correlated fading amplitudes. Proceedings of the 2008 4th IEEE International Conference on Circuits and Systems for Communications.

[16] Wang J., Shen Z. An improved music toa estimator for rfid positioning. Proceedings of the 2002 International Radar Conference.

[17] Kim S., Oh D., Lee J. (2015). Joint dft-esprit estimation for toa and doa in vehicle fmcw radars. IEEE Antennas Wirel. Propag. Lett..

[18] Ding R., Qian Z.-H., Wang X. (2010). Uwb positioning system based on joint toa and doa estimation. J. Electron. Inf. Technol..

[19] Ba B., Liu G.-C., Li T., Lin Y.-C., Wang Y. (2015). Joint for time of arrival and direction of arrival estimation algorithm based on the subspace of extended hadamard product. Acta Phys. Sin..

[20] Jiang H., Wang S.-X. On temporal smoothing for two-dimensional direction-of-arrival estimation of coherent signals in multiplicative/additive noises environment. Proceedings of the 2005 Asia-Pacific Conference on Communications.

[21] Xu H., Wang D., Ba B., Cui W., Zhang Y. (2019). Direction-of-arrival estimation for both uncorrelated and coherent signals in coprime array. IEEE Access.

[22] Du J., Cui W., Ba B., Jian C., Li H. (2022). Fast joint estimation for time delay and angle of arrival based on smooth preprocessing with orthogonal frequency division multiplexing. Iet Radar Sonar Navig..

[23] Lin Y., Jin S., Matthaiou M., You X. (2021). Channel estimation and user localization for irs-assisted mimo-ofdm systems. IEEE Trans. Wirel. Commun..

[24] Ma T., Xiao Y., Lei X., Xiong W., Ding Y. (2021). Indoor localization with reconfigurable intelligent surface. IEEE Commun. Lett..

[25] Li X., Cui W., Xu H., Ba B., Zhang Y. (2020). A novel method for doa and time delay joint estimation in multipath ofdm environment. Int. J. Antennas Propag..

[26] Kolda T.G., Bader B.W. (2009). Tensor decompositions and applications. SIAM Rev..

[27] Jakobsen M.L., Laugesen K., Manchón C.N., Kirkelund G.E., Rom C., Fleury B. Parametric modeling and pilot-aided estimation of the wireless multipath channel in ofdm systems. Proceedings of the 2010 IEEE International Conference on Communications.

[28] Du W., Kirlin R.L. (1991). Improved spatial smoothing techniques for doa estimation of coherent signals. IEEE Trans. Signal Process..

[29] Stoica P., Nehorai A. (1989). Music, maximum likelihood, and cramer-rao bound. IEEE Trans. Acoust. Speech Signal Process..

